# Analysis of the clinical factors associated with anal function after intersphincteric resection for very low rectal cancer

**DOI:** 10.1186/1477-7819-11-24

**Published:** 2013-01-28

**Authors:** Tadao Tokoro, Kiyotaka Okuno, Jin-ichi Hida, Kazuki Ueda, Tahehito Yoshifuji, Koji Daito, Masako Takemoto, Fumiaki Sugiura

**Affiliations:** 1 Department of Surgery, Kinki University, Faculty of Medicine, 377-2, Ohno-Higashi, Osaka-Sayama, Osaka, 589-8511, Japan

**Keywords:** Intersphincteric resection, Very low rectal cancer, Wexner score

## Abstract

**Background:**

Intersphincteric resection (ISR) has been used to avoid permanent colostomy in very low rectal cancer patients. This study aimed to assess the surgical safety and oncologic and functional outcomes of ISR.

**Methods:**

The records of 30 consecutive very low rectal cancer patients who underwent ISR without neoadjuvant therapy were retrospectively analyzed; survival and locoregional recurrence rates were calculated by the Kaplan-Meier method. Incontinence was assessed by a functionality questionnaire and the Wexner score.

**Results:**

The median distance between the distal margin of the dentate line was 10 mm. A total of 12, 4, and 14 patients underwent partial ISR, subtotal ISR, and total ISR, respectively. The mean distal resection margin was negative in all cases, and circumferential resection margin was positive in two cases. Morbidity was 33.3%: anastomotic stricture in seven patients, colonic J-pouch prolapse in two patients, and an anovaginal fistula in one patient. During the median, 56.2-month follow-up period, local, distant, and combined recurrences occurred in four, three, and two patients, respectively. The 5-year overall and disease-free survival rates were 76.5% and 68.4%, respectively. Local recurrence rates were 5.2% for the patients with Tis-T2 tumors as compared with 45.5% for those with T3 tumors (*P* = 0.008). The mean Wexner scores and stool frequencies, 12 months after stoma closure in 19 patients, were 11.5 and 6.6 per 24 h, respectively. Significant differences were not seen in the Wexner scores between partial ISR and subtotal/total ISR (11.8 ± 2.6 and 9.1 ± 5.6). Stool frequency (*P* = 0.02), urgency (*P* = 0.04), and fragmentation (*P* = 0.015) were worse in patients with anastomotic stricture than in those without; there was no symptom improvement in patients with anastomotic stricture.

**Conclusions:**

The anastomotic strictures in patients undergoing ISR may have negatively affected anal function. For total ISR patients, at least, informed consent stating the possibility of a permanent colostomy is necessary.

## Background

Over the last two decades, surgical treatment for patients with very low rectal cancer has radically evolved, allowing permanent colostomy to be avoided in these patients. Reappraisal of the distal margin has allowed increased potency of sphincter-preserving resections. Moreover, total mesorectal excision (TME) [1], coupled with techniques such as end-anal stapling and coloanal anastomosis using the double-stapling technique (DST) [2], can be used to preserve the sphincter without compromising on the oncological results [3-5].

However, when the tumor is located close to the dentate line, conventional anterior resection using the interperitoneal approach with DST may not allow a secure distal resection margin. To resolve this problem, partial or total internal sphincteric resection (ISR) and coloanal anastomosis per anus can be used for safe surgical resection of the tumor [6-11]. ISR has been proposed to achieve distal clearance in selected patients with very low rectal tumors extending to the upper part of the internal sphincter muscle. Furthermore, it has been proposed to restore the anal structure, preserve fecal continence, and reduce the numbers of patients requiring a permanent stoma.

ISR has been widely recognized to achieve a safe distal resection margin, which can be as small as 1 to 2 cm [12,13]. However, with this procedure, which involves dividing the rectum between the internal sphincter and the external sphincter or the levator ani, it remains unclear whether a secure circumferential resection margin (CRM) of the tumor can be obtained. Further, partial or total ISR procedures have been shown to possibly interfere with fecal continence [7,8,14-16].

Anal incontinence is considered to influence various factors in patients receiving ISR, including preoperative radiation therapy [17,18], reconstruction methods [9], extent of sphincter preservation [19], tumor level, and height of the anastomosis [20]. Moreover, fecal incontinence-related quality of life (QOL) scores were poorer in ISR patients than the patients with low anterior resection [16]. Although ISR was proposed as an alternative procedure to avoid abdominoperineal resection (APR), a colostomy is a viable option for patients who suffer from fecal incontinence, which offers a definitive cure along with an improved quality of life [21].

To evaluate the feasibility of ISR in very low rectal cancer patients, it is necessary to clarify the oncologic results and functional outcomes related to this procedure. The aims of this study were to evaluate the surgical safety of the procedure, to assess its oncologic and functional outcomes, and to identify factors predictive of anal dysfunction in the absence of radiotherapy.

## Methods

### Patients

We reviewed the medical charts of all 30 consecutive patients who had undergone ISR for very low rectal adenocarcinoma between April 2001 and August 2010 at the Department of Surgery, Faculty of Medicine, Kinki University. Written informed consent forms concerning this procedure were obtained for all patients in our hospital. In all cases, tumor stage was evaluated before surgery by digital examination; colonoscopy; chest, abdominal and pelvic computed tomography (CT); and pelvic magnetic resonance imaging (MRI). Anorectal manometry was not routinely performed. Preoperative criteria for the exclusion of patients for ISR were clinical T4 tumors, poorly differentiated adenocarcinoma (revealed by biopsy specimens), infiltrating gross appearance of the tumors, and some degree of preoperative incontinence. Among patients with T1 tumors considered for transanal local excision, ISR was proposed for those patients with a risk of lymph node metastases in the case of tumors with adverse pathologic features. Resectable distant metastases were not a preoperative exclusion criterion for ISR, and therefore, ISR was performed in one patient with synchronous liver metastasis.

The histopathological findings and tumor stage classification were based on the Union for International Cancer Control (UICC) TNM classification (seventh edition) [22]. In Japan, preoperative chemoradiotherapy (CRT) for resectable T3 rectal tumors, irrespective of lymph node involvement, was not routinely performed, and none of the patients included in this study had received preoperative CRT or pre or postoperative radiotherapy.

### Surgical technique

The principle of the ISR procedure is based on an anatomic dissection plane between the internal sphincter muscle, which is an extension of the muscular layer of the rectum, and the external sphincter muscle. Surgical intervention was commenced with a high ligation of the inferior mesenteric artery using the abdominal approach. The rectum was dissected to the levator ani with TME. Further, the intersphincteric plane was entered from the nearest anorectal junction if possible. If this dissection was technically difficult to perform until a sufficient distal margin was obtained via the abdominal approach, then the transanal approach of the operation was commenced after perineal exposure using a retractor (Lone Star retractor, Lone Star Medical Products Inc, Houston, TX, USA). The distal margin was 1 cm for Tis-T2 tumors, and 2 cm below the inferior extent of the tumor for T3 tumors. Total ISR involved complete excision of the internal sphincter muscle, that is, the distal line of resection was along the intersphincteric groove. For partial ISR, the distal resection line was along the dentate line, and for subtotal ISR, the distal resection line ran from the dentate line to intersphincteric groove [11,19]. If the tumor was close to the external sphincter or the levator ani muscle, additional partial external sphincter resection (ESR) [11] was performed.

The proximal rectal side of the cut edge was immediately closed and irrigated with 1,500 ml of a 5% povidone-iodine solution to reduce the risk of tumor-cell dissemination [7,23]. Then, the dissection was carried out longitudinally along the plane between the internal and external sphincters to reach the abdominal excision. After the rectum was removed through the abdomen, colonic J-pouch and anal anastomosis procedures with interrupted suture were performed. The anastomosis was protected with a diverting loop ileostomy or transverse colostomy in all the patients.

### Follow-up and local recurrences

All 30 patients were followed for a median of 56.2 months (range; 13.3 to 168.4 months), and 20 patients were available for follow-up for more than 2 years. All patients were followed using a standardized protocol, including a clinical examination with digital palpation, and laboratory tests, including tumors markers (carcinoembryonic antigen (CEA), CA-19-9), every 3 months for the first 3 years, and then every 6 months for 2 years, and then once a year. Abdominal and pelvic computed tomography and chest radiography were performed every 6 months for the first 3 years. A colonoscopy was performed 3 or 6 months after surgery for planning stoma closure, and then once every year for 3 years. Most patients with stage III rectal cancer received postoperative chemotherapy with oral tegafur, uracil, and/or folic acid for 6 to 12 months. Local recurrence was defined as the presence of any anastomotic, pelvic, or lateral node recurrences documented either by clinical or pathologic exanimation, irrespective of the presence of distant metastases.

### Anal functional assessments

Functional outcomes were assessed using our functional questionnaire. We prospectively collected questionnaires regarding anal function from our patients every 3 months after closure of the diverting stoma. In this questionnaire, patients were asked about stool frequency (number of bowel movements per 24 h), fecal urgency (ability to defer stool evacuation for >15 minutes), stool fragmentation (>2 evacuations in 1 h), dyschesia (taking more than 15 minutes to defecate), nocturnal defecation, use of intestinal transit regulators, and need to wear a pad. Incontinence was assessed by the Wexner continence score [24], and we considered anal function to be poor if the Wexner score was 15 or more at 12 months [17,18]. Anastomotic stricture or occlusion was determined when the surgeon’s forefinger could not pass through the anastomotic site 3 months after surgery.

### Statistical analysis

Statistical analyses were performed using JMP10 software (SAS Institute Inc., Cary, NC, USA). Overall and disease-free survival were analyzed using Kaplan-Meier curves and the log rank test. For disease-free survival, patients who failed locally, systemically, or both were censored at the time of the first failure.

Univariate and multivariate regression analyses were used to evaluate the impact of age, gender, type of surgery, type of reconstruction, and anastomotic stricture. The changes in anal function between the different groups of patients over time were compared using Wilcoxon signed-rank test, and comparisons between the anastomotic stricture group and the non-stricture groups were performed using the Mann–Whitney U test. Statistical significance was indicated at the *P* <0.05 level.

## Results

Patients and tumor characteristics are shown in Table 1.

**Table 1:** Clinicopathological characteristics of patients who received intersphincteric resection (n = 30)

**Characteristic**	**Value**
Age, years^a^	60.5 ± 9.9
Histopathological grade^b^	
G1	12
G2	16
Muc	2
Tumor location	
Anterior wall	14
Posterior wall	12
Left wall	1
Right wall	2
Circ	1
Tumor size, cm^a^	3.8 ± 1.5
<4 cm	18
≥4 cm	12
pT stage	
Tis	1 (3.3%)
T1	8 (26.7%)
T2	10 (33.3%)
T3	11 (36.7%)
TNM stage	
0	1
I	16
IIA	5
IIIA	2
IIIB	5
IVA	1

^a^Values denote mean ± SD.

^b^Differentiation of adenocarcinoma: G1 = well differentiated; G2 = moderately differentiated; Muc = mucinous carcinoma. Circ = circumferential tumor.

During the study period, ISR covered 144 patients (26.3%) who underwent surgery for lower-third rectal cancer, located below the peritoneal reflex, 49 patients of conventional anterior resection with DST, 35 patients of abdominoperineal resection, and 20 patients of local excision. The study population was made up of 30 patients (16 men and 14 women) with a median age of 58.9 years (range, 31 to 75 years); 1 patient (3.3%) had a pTis of a large villous tumor, 8 patients had a pT1 tumor (26.7%), 10 patients had a pT2 tumor (33.3%) and 11 patients had a pT3 tumor (36.7%). According to the UICC TNM classification system, the tumors were classified as stage 0 in 1 patient, stage I in 16 patients, stage IIA in 5 patients, stage IIIB in 5 patients, and stage IVA in 1 patient.

Surgical and histopathological findings are shown in Table 2.

**Table 2:** Differences in clinicopathological characteristics between intersphincteric resection (ISR) procedures

	**Partial ISR, (n = 11)**	**Subtotal ISR, (n = 4)**	**Total ISR, (n = 14)**
Sex			
Male	6	2	6
Female	6	2	8
Type of reconstruction			
Colonic J-pouch	9	4	13
Straight	3	0	1
Combined with partial ESR	0	2	2
Distance between the distal edge of the tumor and the dentate line, mm^a^	16.0 ± 4.6	5.0 ± 4.1	3.5 ± 5.1
Distal resection margin, mm^a^	8.7 ± 6.0	9.5 ± 10.5	7.2 ± 5.4
CRM, mm^a^	3.2 ± 2.7	4.8 ± 3.1	3.6 ± 2.1
No. of stoma closures^b^	9 (81.8)	2 (50)	8 (57.1)

^a^Values indicate mean ± SD.

^b^Data in parentheses represent percentage values in each group.

CRM = circumferential resection margin; ESR = external sphincteric resection; ISR = intersphincteric resection.

In this study, partial ISR, subtotal ISR, and total ISR were performed in 12, 4, and 14 patients, respectively. Furthermore, 4 of 11 patients (36.4%) with T3 tumors intraoperatively decided to undergo additional partial ESR. The mean distance between the distal edge of the tumor and the dentate line was 8.9 ± 8.0 mm (range, -3 to 25 mm) in all the patients. Tumor location was significantly different for each ISR procedure (partial ISR, 16.0 ± 4.6 mm; subtotal ISR, 5.0 ± 4.1 mm; total ISR, 3.5 ± 5.1 mm).

Assessment of the fixed surgical specimens revealed that the median distal edge of the tumor was 7 mm (range, 3 to 22 mm), and it was negative in all cases. The median circumferential margin of the tumor was 3 mm (range, 0.5 to 9 mm). The circumferential resection margin was positive (<1 mm) in two patients with T3 tumor without partial ESR. Reconstruction of the colonic J-pouch was performed in 26 patients, and straight coloanal anastomosis was performed in 4 patients due to narrow pelvis or bulky mesocolic fat tissue.

### Mortality and morbidity

There was no mortality. Complications were encountered in ten patients (33.3%). Anastomotic leakage occurred in seven patients, who were treated with perianal drainage. The colonic J-pouch prolapsed in two patients who underwent total ISR. One patient had an anovaginal fistula, requiring repair of fistula using perineal muscular rotation flap, and subsequent stoma closure. Anastomotic stricture or complete occlusion of an anastomosis occurred in seven patients. Of these seven patients, five patients required dilation of the anastomosis using finger bougie, endoscopic balloon dilation, or surgical stricture plasty before stoma closure. Two patients suffered complete occlusion of the anastomosis.

### Oncologic results

Local, distant, and combined recurrence occurred in four, three, and two patients, respectively. Six patients died of cancer recurrence. For all patients who received ISR, the 5-year overall and disease-free survival rates were 76.5% and 68.4%, respectively.

**Figure 1:**
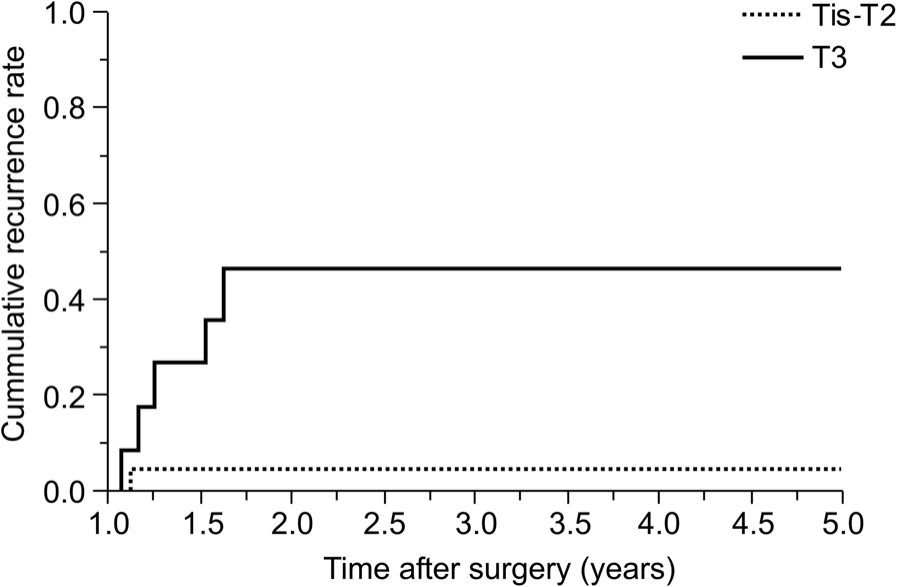
Rates of local recurrence among 30 patients undergoing intersphincteric resection according to the pathologic depth of the tumors (T stage).

The median disease-free interval for six patients with local recurrence was 13 months (range, 8 to 14 months) (Table 3). All of the four isolated local recurrence episodes developed within the first 2 years. All the patients who experienced local recurrence had pT3 tumors, except one patient who had a pT2 tumor. The local recurrence rates were significantly lower in patients with Tis to T2 tumors (5.2%) than in those with T3 tumors (45.5%; *P* = 0.008; Figure 1).

**Table 3:** Characteristics of six patients with local recurrence after intersphincteric resection (ISR)

**Patient**	**TNM**	**T stage**	**Histological type**	**Surgical procedure**	**Distal resection margin, mm**	**Circumferential resection margin, mm**	**Localization**	**Distant metastases**	**Treatment**	**Outcome**
1	IIA	T3	G2	tISR + pESR	7	2	Pelvic wall	NS	CRT	45 months, O
2	IIIB	T3	G2	sISR + pESR	25	2	Pelvic wall	NS	CRT	70 months, S
3	IIIB	T3	G2	pISR	12	5	Pelvic wall	Bone, lung	Cx	31 months, P
4	IIA	T3	G2	pISR	10	0.5	Lateral node	Adrenal gland	Cx	36 months, P
5	I	T2	G1	pISR	3	6	Anastomosis	NS	APR	22 months, S
6	IIB	T3	G1	pISR	3	0.5	Lateral node	NS	CRT	17 months, S

Distal resection margins and circumferential resection margins were measured on the histological slides.

APR = abdominoperineal resection; CRT = chemoradiotherapy; Cx = chemotherapy; ESR = external intersphincteric resection; ISR = intersphincteric resection; NS = not stated; O = other origin of death; P = primary death; S = survived.

### Aspects of stoma closure

Of the 29 ISR patients, excluding 1 with stage IVA disease, 19 (65.5%) underwent stoma closure by February 2010, including 3 patients who had undergone straight anastomosis. The median interval between ISR and stoma closure was 7 months (range, 3 to 14 months). The median follow-up interval after stoma closure was 35 months (range, 4 to 68 months). Nine, two, and eight patients received stoma closure in the partial ISR, subtotal ISR, and total ISR groups, respectively (Table 2).

Definitive stoma closure could not be performed in 11 patients. Of the 11 patients, 5 had insufficient anal condition (complete anastomotic occlusions in 2, prolapse of colonic J-pouch in 2, obvious loose anastomosis in 1). The patients who developed colonic J-pouch prolapse or obvious loose anastomosis had received total ISR. Four patients were diagnosed with distant metastases or local relapse of the disease before stoma closure. Two patients did not undergo stoma closure for social reasons. Three out of four patients with additional partial ESR did not achieve stoma closure because of a colonic J-pouch prolapse or local recurrence.

### Evaluation of anal function

Anal function was evaluated in 19 patients who underwent stoma closure. At 12 months after stoma closure, the mean Wexner score for all patients was 11.5 (range, 1 to 19). In the patients with partial ISR, the Wexner scores were improved from 13.0 ± 3.1 at 3 months to 12.1 ± 3.0 at 6 months (*P* = 0.04). In contrast, in the patients with subtotal or total ISR, no significant differences were found between the Wexner scores at 3 months and 6 months (13.0 ± 3.8 and 11.5 ± 4.9, respectively; *P* = 0.14), but an upward trend was observed in the Wexner scores at 6 months and 12 months (11.5 ± 4.9 vs 9.1 ± 5.6, respectively, *P* = 0.06). At 3, 6, and 12 months, the Wexner scores were not significantly different between patients who underwent partial and subtotal or total ISR (Table 4). In the patients without anastomotic stricture, the Wexner scores were significantly improved at 6 months and 12 months compared with those at 3 months. However, five patients, including the one with subtotal ISR and an additional partial ESR, required finger bougie, endoscopic balloon dilation, or stricture plasty for anastomotic stricture, no improvement in the Wexner score was observed.

**Table 4:** Wexner scores at 3, 6, and 12 months in patients who underwent intersphincteric resection (ISR) followed by stoma closure

**Procedure/findings**	**3 months**	**6 months**	**12 months**
Surgical procedure			
Partial ISR (n = 9)	13.3 ± 3.1	12.1 ± 3.0*	11.8 ± 2.6
Subtotal or total ISR (n = 10)	13.0 ± 3.8	11.5 ± 4.9	9.1 ± 5.6
Anastomotic stricture			
Yes (n = 5)	15.4 ± 2.9	13.4 ± 4.5	13.6 ± 3.9
No (n =14)	12.4 ± 3.3	11.2 ± 3.9*	9.0 ± 4.5*

Data are shown as mean ± SD. Data at 6 and 12 months were statistically compared with those at 3 months using the Wilcoxon signed-rank test.

*P <0.05.

Table 5 shows the anal function based on the questionnaires answered at 3, 6, and 12 months after stoma closure, with or without anastomotic stricture. At 12 months after stoma closure, patients without anastomotic stricture were showed improved urgency (from 12/14 to 3/12; *P* = 0.008) and nocturnal defecation (from 9/14 to 5/12; *P* = 0.014). However, patients with anastomotic stricture did not report improvement in any symptom. Compared to patients with anastomotic stricture, the non-stricture group showed significantly better results with regard to stool frequency (5.1 ± 2.9 vs 9.0 ± 5.3; *P* = 0.02), urgency (3/12 vs 4/5; *P* = 0.04), and fragmentation (4/12 vs 5/5; *P* = 0.015) at 12 months.

**Table 5:** Anal dysfunction after stoma closure in patients with anastomotic stricture and those with no anastomotic stricture

**Symptoms related to anal function**	**3 months**		**6 months**		**12 months**	
	**Non-stricture**	**Stricture**	**Non-stricture**	**Stricture**	**Non-stricture**	**Stricture**
Stool frequency, times/day	6.4 ± 3.6	11.4 ± 3.0**	6.8 ± 2.8	7.0 ± 2.1	5.1 ± 2.9	9.0 ± 5.3**
Urgency	12/14	4/5	8/13	2/5	3/12*	4/5**
Fragmentation	10/14	5/5	7/13	4/5	4/12	5/5**
Dyschesia	2/14	3/5	2/13	0/5	2/12	0/5
Medication use	4/14	4/5	3/13	4/5**	2/12	3/5
Nocturnal defecation	9/14	5/5	5/13*	5/5	5/12*	4/5

Data associated with each anal dysfunction at 6 and 12 months were statistically compared with those at 3 months for each condition of anastomotic sites using Wilcoxon signed-rank test. *P <0.05.

Data associated with each anal dysfunction of the stricture group were statistically compared with those of the non-stricture group at 3, 6, and 12 months using the Mann–Whitney U test. **P <0.05.

The results of the univariate analysis revealed that poor anal function, as assessed by the Wexner score, was significantly associated with gender (male; *P* = 0.047) and the presence of anastomotic stricture (*P* = 0.018) at 12 months. The surgical procedure (partial or subtotal/total ISR), type of reconstruction (straight or colonic J-pouch), and age (<70 or ≥70) were not significantly associated with anal function. The results of the multivariate analysis also showed that gender (*P* = 0.283) was not significantly associated with anal function and that the presence of anastomotic stricture (*P* = 0.093) only demonstrated a trend towards being significantly associated with anal function (data not shown).

## Discussion

Although ISR is the sphincter-preserving procedure for very low rectal cancer, there are concerns regarding local control and defecatory function. In this study, we report the outcomes of ISR of very low rectal cancer, less than 2.5 cm from the dentate line, with a median follow-up period of 56 months. Our data show that this operation is feasible, with no postoperative mortality found in the study group. Moreover, it is associated with favorable oncological outcomes for Tis-T2 tumors. With regard to the Wexner score, total ISR did not produce worse outcomes than partial ISR did, with the exception that permanent stoma were necessitated by unfavorable anastomosis. However, anastomotic stricture, which occurred as a postoperative complication, was found to negatively affect anal function.

From an oncological point of view, local control of the disease remains the most important objective in rectal cancer surgery. The local recurrence rate of very low rectal cancer for ISR varied widely, ranging between 0% [23] to 31% [25]. With ISR, the rate of secure distal resection margin was in the range of 95% [23] to 100% [18], and our results showed a median distance of 7 mm, and a definite negative distal margin in all patients. Therefore, ISR was found to provide an optimal distal resection margin, which is difficult to attain by using only the abdominal approach for very low rectal cancer. Rate of positive CRM of the rectal cancer also influenced factor of local recurrence. In our study group, 6.7% of all patients had a CRM ≤1 mm, and similar results were reported in the range of 0% to 13.3% [18,26]. Preoperative CRT was considered useful for preventing local recurrence in low rectal cancer patients requiring ISR [14,23]. Kuo *et al*. [26] reported a positive CRM rate of 13.3%, but a local recurrence rate of 7.7% in their ISR series of 26 patients; 88.5% of these patients had undergone preoperative CRT. Paradoxically, Hohenberger and colleagues [27] reported that in ISR patients with lower-third rectal cancer without radiotherapy, the local recurrence rate was high, at 46.5%. In our study, local recurrence was significantly higher in patients with T3 tumors than in those with Tis-T2 tumors. Akasu *et al*. [28] reported that both T3 tumors and a positive microscopic resection margin in patients who underwent ISR were significantly associated with local recurrence. Because ISR involves dissection of the rectum between the internal sphincter muscle and the external sphincter muscle, in patients with T3 tumors with expanding microscopic tumor cells near the levator ani or the external sphincter muscles, during surgical resection, there is a considerable risk of cutting into the tumor or achieving a very short distance of a few millimeters to the CRM. Thus, for a group of patients with T3 tumors, ISR was applied to attain good responses to neoadjuvant CRT, leading to secure CRM.

Partial or total resection of the internal sphincter muscle resulted in defecatory dysfunction with frequent defecation, urgency, and fecal incontinence [16,18,29]. Moreover, preoperative radiotherapy against T3 tumors or lymph node involvement was found to have a negative impact on anal function after ISR [17,18,26]. In the study by Ito *et al*. [17], of all the patients who underwent ISR, 40% received radiotherapy and were found to have a mean Wexner score of 10 at 12 months. Moreover, Denost *et al*. [20] reported a median Wexner score of 11 in most of the patients who received radiotherapy.

It has been shown that colonic J-pouch reconstruction in conjunction with ISR can minimize the anal dysfunction-related side effects of a sphincteric resection [9]. Hida *et al*. [30] reported the long-term benefits of colonic J-pouch reconstruction suggesting that it improves reservoir function to a greater extent than straight anastomosis does, especially in patients in whom the anastomosis is less than 4 cm from the anal verge. In addition, Dennett *et al*. [31] reported that colonic J-pouch is effective in very low rectal cancer surgery, causing apparent reduction in the incidence of anastomotic leaks and in bowel frequency. In our study, the mean Wexner score was 11.5 in most patients with colonic J-pouch reconstruction, and none of the patients had received radiation therapy. In previous studies, total ISR was performed in 8.9% [29] to 33.7% [20] of all the ISR patients. A possible reason for the poorer outcomes about Wexner score in our study was that the number of patients who required total ISR accounted for approximately half the ISR patients (42.1%), because coloanal anastomosis using conventional DST was technically possible in a few patients who required partial ISR during our study period.

The outcome for continence is reported to be worse after total ISR than after subtotal or partial ISR [19,20]. In our results for Wexner scores, anal function between total or subtotal ISR and partial ISR were not different, but patients of partial ISR had earlier recovery than those of subtotal or total ISR. Our functional results are limited because of the differences in stoma closure rates between partial ISR and subtotal/total ISR patients. The rates of stoma closure in patients with subtotal or total ISR were lower than those in patients undergoing partial ISR. This result in itself indicates poor anal function outcomes for subtotal/total ISR. Especially with respect to the three patients with total ISR, stoma closure was not possible because of the high risk of major incontinence.

Postoperative complication rates varied between reported series from 18% to 64% [15]. Common complications included leakage, anastomotic stricture, fistula, pelvic sepsis, and prolapse. In a previous literature review, anastomotic leakage rates of 5% to 48% [32] were reportedly associated with ISR, and they varied depending on whether asymptomatic leaks were radiologically detected. Also, Tilney and Tekkis [9] reviewed 21 studies and reported an overall anastomotic leak rate of 10.5% and anastomotic stricture rate of 5.8%. Similar rates were reported in the current series: anastomotic leakage occurred in 7 of 30 patients (23.3%) and anastomotic stricture in 12% of the patients. Anastomotic leakage is an important feature since it has been found to lead to postoperative anastomotic stricture [33] and poor postoperative anorectal function [34]. However, in our study, there were no independent factors associated with anal dysfunction in the multivariate analysis, but patients with anastomotic stricture showed worse outcomes (frequency, urgency, and fragmentation) than patients without anastomotic stricture. In addition, symptoms related to anal function were not reduced in these patients. In our study, anastomotic stricture or occlusion occurred in five of seven patients with anastomotic leakage; thus, stricture formation could be attributed to leakage caused by ischemia or infection of the anastomotic site. Therefore, it is necessary to fully explain the possibility of fecal incontinence or of a permanent stoma to the patients before obtaining informed consent. Fecal QOL in our patients who had an anastomotic stricture was worse, and they might have little benefit from preserving the anal continuity with ISR.

Our study has some limitations: it was a retrospective study and the sample size was relatively small. There could be potential bias due to possible difference between those who were ambitious of receiving the anal sphincter preserving surgery and those who did not, which could affect the self-evaluation for gastrointestinal questionnaire. With regard to the additional partial ESR performed only in one patient with stoma closure, this was not taken into consideration while estimating anal function.

## Conclusions

In summary, ISR is an oncologically safe procedure for pTis or pT2 tumors among very low rectal cancer patients. Also, total ISR, that is, complete removal of the internal sphincter muscles, carried risks of worse anal function or possibility of a permanent stoma. The complications associated with anastomosis, especially stenosis, resulted in poorer anal function. Larger studies are needed to evaluate functional results in ISR patients who suffer from anastomotic stricture.

## Abbreviations

CRM: circumferential resection margin; DST: double-stapling technique; ESR: external sphincter resection; ISR: intersphincteric resection; TME: total mesorectal excision.

## Competing interests

The authors declare they have no competing interests.

## Authors’ contributions

TT, KO, JH, KU, TY, KD, MT, FS: made substantial contributions to conception and design, and/or acquisition of data, and/or analysis and interpretation of data. TT, KO, JH: drafted the article and revised it critically for important intellectual content. TT, KO, JH, KU, TY, KD, MK, FS: responsible for final approval of the manuscript. All authors read and approved the final manuscript.
